# Personal

**Published:** 1896-12

**Authors:** 


### Personal.

Hon. Wm. P. Letchworth, for twenty-three years a member of the state board of charities, has resigned his office, and Mr. Harvey W. Putnam, of Buffalo, has been appointed to fill the vacancy. Mr. Letchworth was president of the board for a number of years, and during the entire period of his membership, no matter in what capacity he has served, he has been a consistent, devoted and able worker in the cause of organised charity, giving his time and money freely for the betterment of the poor, the insane and even the criminal. We hope his advice will still be available to the state, even though he has felt compelled to retire from active labor. Mr. Letchworth's whole life has been an exemplification of the precept, "And the greatest of these is charity."

Mr. Putnam, who succeeds Mr. Letchworth, is well known in this city as a lawyer and as secretary of the civil service commission. By birth and education, as well as by training during his young manhood, he has become well prepared for the work before him, and we wish him all the success that distinguished his predecessor. We could not say more.

---

Dr. William B. May, of Buffalo, formerly of the police force, a graduate of Buffalo University Medical College at the last annual commencement, has established his office at 847 Clinton street. Dr. May was a popular and efficient officer and acquired his professional training in service. He deserves success in his profession and no doubt will attain it.

---

Hon. Timothy L. Woodruff, president of the Maltine Manufacturing Company, has been elected lieutenant-governor of the state of New York by the phenomenal majority of a quarter of a million of votes. Mr. Woodruff is at present park commissioner of Brooklyn, and is a man of affairs, trained to thought and action, a graduate of Yale, and is every way fitted by birth, education and wealth to adorn the great office for which he has been chosen. Lieutenant-Governor and Mrs. Woodruff will gracefully adorn the official and social life of Albany for the next two years.

---

Dr. Jacob Miller, of Buffalo, will visit Europe early in January, 1897, for professional advancement and recreation. He will spend some time in the clinic of Lepold, at Dresden, and will also stay a few weeks at Berlin, where he will take a course in pedriatics. Dr. Miller will remain abroad about six months.

---

Dr. Byron H. Daggett, of Buffalo, has removed from 258 Franklin street to 2732 Main street. His office is located at 847 Ellicott Square. Hours : 11-1, 2-4 and 7 p. m.

